# Influences of earthworm activity and mucus on Cd phytoremediation based on harvesting different leaf types of tall fescue (*Festuca arundinacea*)

**DOI:** 10.1371/journal.pone.0304689

**Published:** 2024-06-14

**Authors:** Hongwei Li, Jie Luo, Min Cao, Wenquan Luo, Xingying Li, Zongqi Shao, Lianming Zhu, Siyao Feng

**Affiliations:** 1 YUNNAN CHIHONG Zn & Ge CO, Ltd., Qujing, China; 2 College of Resources and Environment, Yangtze University, Wuhan, China; 3 University of Leicester, Leicester, United Kingdom; Universidade de Coimbra, PORTUGAL

## Abstract

To explore cost-effective and efficient phytoremediation strategies, this study investigated the distinct roles of earthworm activity and mucus in enhancing Cd phytoextraction from soils contaminated by *Festuca arundinacea*, focusing on the comparative advantages of selective leaf harvesting versus traditional whole-plant harvesting methods. Our study employed a horticultural trial to explore how earthworm activity and mucus affect *Festuca arundinacea*’ s Cd phytoremediation in soils using control, earthworm, and mucus treatments to examine their respective effects on plant growth and Cd distribution. Earthworm activity increased the dry weight of leaves by 13.5% and significantly increased the dry weights of declining and senescent leaves, surpassing that of the control by more than 40%. Earthworm mucus had a similar, albeit less pronounced, effect on plant growth than earthworm activity. This study not only validated the significant role of earthworm activity in enhancing Cd phytoextraction by *Festuca arundinacea*, with earthworm activity leading to over 85% of Cd being allocated to senescent tissues that comprise only approximately 20% of the plant biomass, but also highlighted a sustainable and cost-effective approach to phytoremediation by emphasizing selective leaf harvesting supported by earthworm activity. By demonstrating that earthworm mucus alone can redistribute Cd with less efficiency compared to live earthworms, our findings offer practical insights into optimizing phytoremediation strategies and underscore the need for further research into the synergistic effects of biological agents in soil remediation processes.

## 1. Introduction

As the economy continues to grow and human material needs increase, a wide range of pollutants are being discharged into soils [1]. Among the different types of pollutant, heavy metal contamination has drawn increasing attention worldwide, especially in developing countries that have long been affected by environmental issues [2, 3]. Based on findings from a nationwide soil survey conducted by Chinese government authorities, approximately 16% of China’s land area is affected by heavy metal contamination, with over 7% specifically affected by Cd [4]. Cadmium is not an essential element for life except in certain microalgae [5]. The toxicity of Cd in different environmental media and living organisms is higher than that of other common metals [6]. Cadmium has detrimental effects on most plants, especially food crops, even at considerably low levels. Farmland pollution by Cd has led to significant economic losses in agricultural nations. This is evident from the decreased yield and compromised food safety of primary agricultural products grown in regions affected by Cd contamination [7]. Human activities, such as the extensive use of chemical fertilizers, have significantly contributed to soil Cd contamination. Studies have shown that phosphate fertilizers, which are often used to enhance soil fertility, can contain high levels of Cd, leading to its accumulation in agricultural soils [8]. In addition to industrial discharge and improper waste management, this poses a severe risk to soil health and food security. The considerable disparity between China’s extensive population and its limited cultivated land presents a formidable obstacle to safeguarding food security. Therefore, there is an urgent need to develop effective soil remediation technologies.

Various physical and chemical methods have been used to address soil contamination [9, 10]. These technologies are suitable for small, heterogeneous sites with high pollution levels. However, these conventional technologies are difficult to use for the amelioration of regional pollution over large areas because of their high cost [11, 12]. The complex topography of some large contaminated fields limits the use of heavy equipment for soil remediation. In addition, some of these conventional soil decontamination methods cause irreversible damage to the fertility, structure, and biotic community of the soil, consequently decreasing the area of arable land [3, 13].

Phytoremediation, a method that employs green plants and their associated microbial communities to mitigate the effects of soil pollutants, is widely recognized as an economically viable and environmentally sustainable solution for addressing metal contamination in cultivated areas [14]. In contrast to other subsets of phytoremediation such as phytostabilization and phytovolatilization, the utilization of plants, particularly hyperaccumulators, for phytoextraction is a more effective means of thoroughly removing pollutants from the soil. This is accomplished by the repositioning and buildup of pollutants in the aboveground portions of plants [15]. Cadmium hyperaccumulators, such as *Noccaea caerulescens* [16], *Arabidopsis helleri* [17], and *Sedum alfredii* [18] are commonly used to remove Cd from soils. However, their low biomass generation ability limits the wide application of hyperaccumulators at the field scale. Furthermore, hyperaccumulators typically mobilize all metals in the rhizosphere soil but selectively accumulate specific metals, potentially posing a risk of leaching of metals that are not hyperaccumulated [19]. Although hyperaccumulators offer a viable solution for phytoextraction, their application is hampered by a low biomass yield and selective metal uptake, which limit their efficiency in large-scale remediation [20]. This highlights a significant gap in employing high-biomass, non-hyperaccumulator plants such as *Festuca arundinacea* (*F*. *arundinacea*) for Cd phytoextraction, underscoring the need for innovative approaches to improve their metal uptake capabilities.

Hence, rapidly growing plants such as tall fescue plants have been used for both phytoremediation and energy generation [21]. Previous studies have documented the robust growth of *F*. *arundinacea* under a range of external conditions, such as drought [22], low fertility, metal pollution [23], and waterlogging [24]. Plant species with high biomass production, such as *F*. *arundinacea*, tend to sequester heavy metals within their belowground tissues under standard environmental conditions [25, 26]. Consequently, various approaches have been used to enhance the metal extraction and translocation capabilities of these plants, including the application of metal-binding agents, microbial introduction, and electrokinetics [27, 28]. However, such supplementary means are too expensive, considering the large expanse of contaminated fields.

Compared with artificial additives, earthworms are ubiquitous invertebrates in the soil that can develop and maintain soil fertility [29]. Earthworms can survive severe pollution even in tailing spoils [30]. The presence of invertebrate species increases the accessibility of metals in the soil and enhances the development of plant roots [31]. Earthworm activity promotes plant growth and increases metal concentrations in plant tissues [32, 33]. These studies indicated that earthworm activity can enhance the phytoextraction efficiency of plants by increasing their biomass yield and metal distribution capacity. Mucus secreted from the body walls of earthworms serves a crucial function in the feeding and defense behaviors of the species. Zhang et al. [34] documented that the mucus of *Metaphire guillemi* stimulates both the growth of *Lycopersicon esculentum* and a significant increase in the Cd content of plant tissues.

The application of *Eisenia andrei* can improve the phytoremediation of *Canavalia ensiformis* for Cu, by increasing the uptake and accumulation capacity of the plant shoots [35]. Unlike previous studies, which primarily explored the physical contributions of earthworms to soil and plant health, we employed earthworm mucus as a stand-in to simulate and isolate the chemical effects of earthworms on the phytoremediation capabilities of *F*. *arundinacea*. It is conceivable that the presence of earthworms and their mucus secretion could enhance the Cd removal capability of the plant by promoting plant development and increasing the Cd availability in the soil. Hence, the primary objectives of this study were to assess the effect of earthworm activity and mucus on (1) changes in leaf biomass and proportion among young, mature, declining, and dead leaves; (2) the distribution of Cd among different leaf types; and (3) the Cd remediation effectiveness of the plant. In an innovative approach to delineate the effects of earthworms on phytoremediation without the variables introduced by their physical activities, we utilized earthworm mucus as a stand-in to simulate the biochemical impact of earthworm presence. This method allowed controlled analysis focusing on the chemical contributions of earthworms to the phytoremediation process. Our results distinctly showed the differential effects of direct earthworm activity (EA) versus earthworm mucus (EM) application, underscoring the enhanced phytoremediation effectiveness observed with actual earthworm activity.

## 2. Materials and methods

### 2.1 Soil collection

The soil was obtained from a town specializing in disassembling and recycling electronic waste. Based on the findings of a previous ecological geochemical assessment, soils within various zones experienced varying degrees of metal contamination [36]. Xu et al. [37] documented that neonates born in this town exhibited elevated blood Cd levels compared to those not associated with electronic waste recycling activities. Therefore, there is an urgent need to safely remediate Cd-contaminated soil distributed in residential districts. Surface soil samples were collected in a 200 × 200 grid at depths of 0–20 cm from residential districts over the entire town to ensure the representativeness of the obtained data.

The soil samples were air-dried on filter paper at room temperature and sieved with 4 mm nylon meshes to eliminate foreign materials. The prepared soil samples were thoroughly blended to create a unified substrate for the subsequent treatments. The composite substrate was subjected to repeated wetting and drying cycles to achieve a uniform distribution of Cd. After each saturation and drying cycle, 20 random soil samples (5 g each) were collected from the substrates for Cd analysis.

To analyze the soil pH, the prepared substrate was blended with deionized water at a ratio of 1:2.5 (w:v), and a pH meter (Hanna Instruments, HI98160, Smithfield, Rhode Island, USA) was used to measure and record the pH of the mixture when the reading stabilized without further alterations [38]. To determine soil exchangeable cations, 33 mL of sodium acetate (CH_3_COONa) was applied to saturate the exchange sites with Na^+^ ions in the soil (2 g), and 33 mL of ammonium acetate (CH_3_COONH_4_) was used to replace Na^+^ with NH_4_^+^. Following centrifugation at 3,000 rpm, the solution underwent filtration through a 0.45 μm pore-size filter [39]. Exchangeable cations in the resulting supernatant were analyzed according to the procedure recommended by Melo et al. [40]. To analyze soil organic carbon (OC), we employed a solution of 0.8 mol L^-1^ potassium dichromate (K_2_Cr_2_O_7_) to oxidize organic substances within the soil. Following oxidation, ferrous sulfate was used for titration [41].

All experimental activities in this study were conducted indoors, and the soil samples were obtained from accessible public domains without the need for special permits. According to the legal and guiding principles of the region, this research does not require specific permits.

### 2.2 Experimental design

In this study, the soil was thoroughly mixed and air-dried, resulting in a relatively loose texture. To ensure uniform soil density across all pots, we measured 6 kg of the prepared soil in 10 cm radius and 20 cm height containers. The soil within each container was compacted to a consistent depth of 16 cm. This procedure was meticulously followed in each pot to maintain experimental consistency. Healthy seeds with a consistent weight and appearance were obtained from a seed supplier (Hanna Instruments, HI98160, Smithfield, Rhode Island, USA). Following surface sterilization using a 2% NaOCl solution, the seeds were cultivated in the sand with an alternating 8-hour night and 16-hour day cycle at temperatures of 18°C and 26°C, according to the growth habit of *F*. *arundinacea* [42]. The moisture content of the sand was maintained at 70% for germination. Following shoot emergence, 40 uniform seedlings were transplanted into pots filled with soil for a 15-day acclimatization period. Afterward, the seedlings were thinned to 30 for further experiments.

Adult *Pheretima guillemi* (*P*. *guillemi*), which can survive multiple metal stresses [43, 44], were bought from a farming factory in Jiangsu Province, China. The initial fresh weight of *P*. *guillemi* was approximately 4.0 g. The earthworms were acclimated to clean soil for a week at 20°C. The acclimated earthworms were rinsed thrice with distilled water, followed by gentle drying using paper tissue to eliminate any excess moisture. Afterward, they were placed in culture dishes resting on damp filter paper. The culture dishes were placed in an incubation box at 20°C for 2 days to thoroughly remove earthworms’ gut contents. The moistened filter paper was replaced three times daily to avoid coprophagy. After the starvation period, a batch of *P*. *guillemi* was washed with distilled water, blotted using paper tissue, and sand cultivated in sterilized glass vessels (30 earthworms per vessel) for 48 h. The earthworms, sand, and vessels were rinsed using deionized water three times after the 48 h cultivation, and the washing water was filtered and kept at -10°C.

The transplanted plants were segregated into three sets of five replicates each. Each pot was irrigated every 3 days using approximately 200 mL of water, adjusted according to the required soil moisture (70%), which was measured using a soil moisture sensor (Yuntang Intelligent Technology Co., Ltd, YT-WS, Shandong, China). Both groups were irrigated with normal water. Earthworms (30 individuals per pot) that had not been subjected to sand cultivation were added to one of the normal water-irrigated groups (EA), whereas the other irrigated group was used as the control (N). The third group was irrigated with 200 mL of washing water (representing mucus) from the sand cultivation procedure (EM) every 3 days. Water was added to the trays beneath each pot to prevent metal leaching.

Plants were harvested 50 d after transplantation. The collected plants were partitioned into aboveground and belowground components. Leaves were categorized as young, mature, declining, and dead. Subsequently, the belowground tissues were washed with tap water to eliminate any adhering soil and thoroughly rinsed with distilled water. To remove any adsorbed ions, the cleaned roots were immersed in a 0.1 mmol L^-1^ CaCl_2_ solution for 30 min. The plant leaves underwent a similar process of initial washing under running water to remove any dust adhering to their surfaces, followed by immersion in a 15 mM Na_2_EDTA solution for 30 min to eliminate adsorbed ions. After ensuring the removal of any residual solution on both the root and leaf surfaces using deionized water, the various plant components were subjected to oven drying at 70°C until a consistent mass was attained.

### 2.3 Soil and plant analyses

The dried samples were finely ground to a particle size of 74 μm using mesh screens. Subsequently, the samples were digested in aqua regia for 2 h [45]. Process that facilitates the extraction of total Cd from soil. After digestion, the solution was diluted with deionized water and passed through a 0.45 μm filter. The concentration of total Cd in the resulting supernatant was quantified using ICP-MS (Agilent Technologies, Agilent 7700, Santa Clara, California, United States).

In this study, the Cd concentration in the soil was assessed at the beginning of the experiment. This was done to verify the uniformity of Cd distribution within the soil, as the duration of this study was insufficient to induce significant changes in the pseudototal Cd content of the composite soil. In line with the findings of Martínez-Alcalá et al. [46], who indicated that *Noccaea caerulescens* (*N*. *caerulescens*), a recognized Cd hyperaccumulator, did not alter the Cd concentration in the target soil over the course of a 56-day experiment, the Cd content in the soil was not assessed at the conclusion of the experiment.

We analyzed the standard reference materials for soil (GBW07410) and plants (GBW10012) provided by the China National Center for Standard Materials alongside every set of 12 samples. The equipment’s detection limit for Cd was determined to be 0.02 mg kg^-1^ in the substrate and 0.03 mg kg^-1^ in *F*. *arundinacea*, respectively.

Some parameters that are commonly employed to evaluate the phytoremediation effectiveness [47, 48] include bioaccumulation factors (BCF) and translocation factors (TF) were computed as follows:

BCF = Cd concentration in plant roots or shoots (mg Cd/kg plant)/Cd concentration in soil (mg Cd/kg soil)

TF = Cd concentration in plant shoots (mg Cd/kg plant)/Cd concentration in plant roots (mg Cd/kg soil).

### 2.4 Statistical analysis

The significance of the treatment effects on leaf biomass, composition of various leaf types, Cd content in plant tissues, and Cd decontamination efficiency was evaluated using one-way analysis of variance (ANOVA). The mean values were compared using Fisher’s least LSD post hoc test, with a significance level of *p* < 0.05. SPSS (version 15.0, SPSS Inc., Chicago, Illinois, USA) was used to perform statistical analyses.

## 3. Results and discussion

### 3.1 Initial soil characteristics and treatment effects

At the outset of the experiment, the initial values for pH, OC, and exchangeable cations in the soil were within the ranges of 5.7 to 6.7, 31.9 to 44.3 g kg^-1^, and 9.6 to 16.8 cmol_c_ kg^-1^, respectively (Table 1). The Cd concentration in the soil ranged from 3.3 to 4.9 mg kg^-1^, with an average of 4.2 mg kg^-1^. The observed Cd content in the soil from the chosen areas significantly surpassed the legally mandated Cd threshold for soil (0.3 mg kg^-1^, China MEP), beyond which toxic effects manifest in plant tissues. Upon completion of the treatment, soil pH in EA, EM, and the control were 6.6 ± 0.7, 6.3 ± 0.5, and 6.0 ± 0.6, respectively. No significant differences were observed relative to the initial pH, indicating that earthworm activity, earthworm mucus, and plants did not change the soil pH in the present study. The highest exchangeable cations were observed in EA (16.3 ± 2.9 cmol_c_ kg^−1^), followed by EM (14.9 ± 4.3 cmol_c_ kg^−1^), and the control (13.7 ± 3.1 cmol_c_ kg^−1^) after the experiment. Compared with the initial value, EA, EM, and the control increased soil OC by 3.9, 12.6, and 9.5%, respectively, probably because of root decay. Despite this increase in OC, no significant deviations were noted in soil pH, exchangeable cation levels, or OC compared to the initial measurements.

**Table 1:** Soil characteristics.

	initial value	N	EA	EM
**pH**	6.2 ± 0.5 a	6.6 ± 0.7 a	6.3 ± 0.5 a	6.0 ± 0.6 a
**OC (g kg** ^**-1**^ **)**	38.1 ± 6.2 a	39.6 ± 5.5 a	42.9 ± 7.6 a	41.7 ± 8.1 a
**Exchangeable cations (cmol**_**c**_ **kg**^**-1**^**)**	13.2 ± 3.6 a	13.7 ± 3.1 a	16.3 ± 2.9 a	14.9 ± 4.3 a
**Cd (mg kg** ^**-1**^ **)**	4.2 ± 0.59 a	-	-	-

N represents for normal condition (control), EA represents for earthworm activities, and EM for earthworm mucus. Alphanumeric characters denote results of Fisher’s LSD post-hoc tests. Tables 1–3 share the same legend.

Hanc and Dreslova [49] and He et al. [50] reported a reduction in soil pH owing to earthworm activity. However, in the current study, a marginal increase in soil pH was found in the EA treatment, although this change was not statistically significant (*p* > 0.05). This discrepancy could be explained by variations in soil characteristics and earthworm species employed [51]. Previous studies have suggested that earthworm activity can improve the structure, density, aeration, and chemical conditions of soil through feeding, casting, excretion, and mucus generation [52, 53]. The results of the present study corroborate those of [54], who observed that earthworm invasion increased exchangeable cations in forest soil; thus, non-native species could be successfully established in fertile soil. These results indicate that earthworm activity (EA) can improve soil quality. Earthworm mucus (EM) produced similar but lower effects on the soil than earthworm activity (EA).

### 3.2 Effects of earthworm activities on *Festuca arundinacea* growth

**Fig 1:**
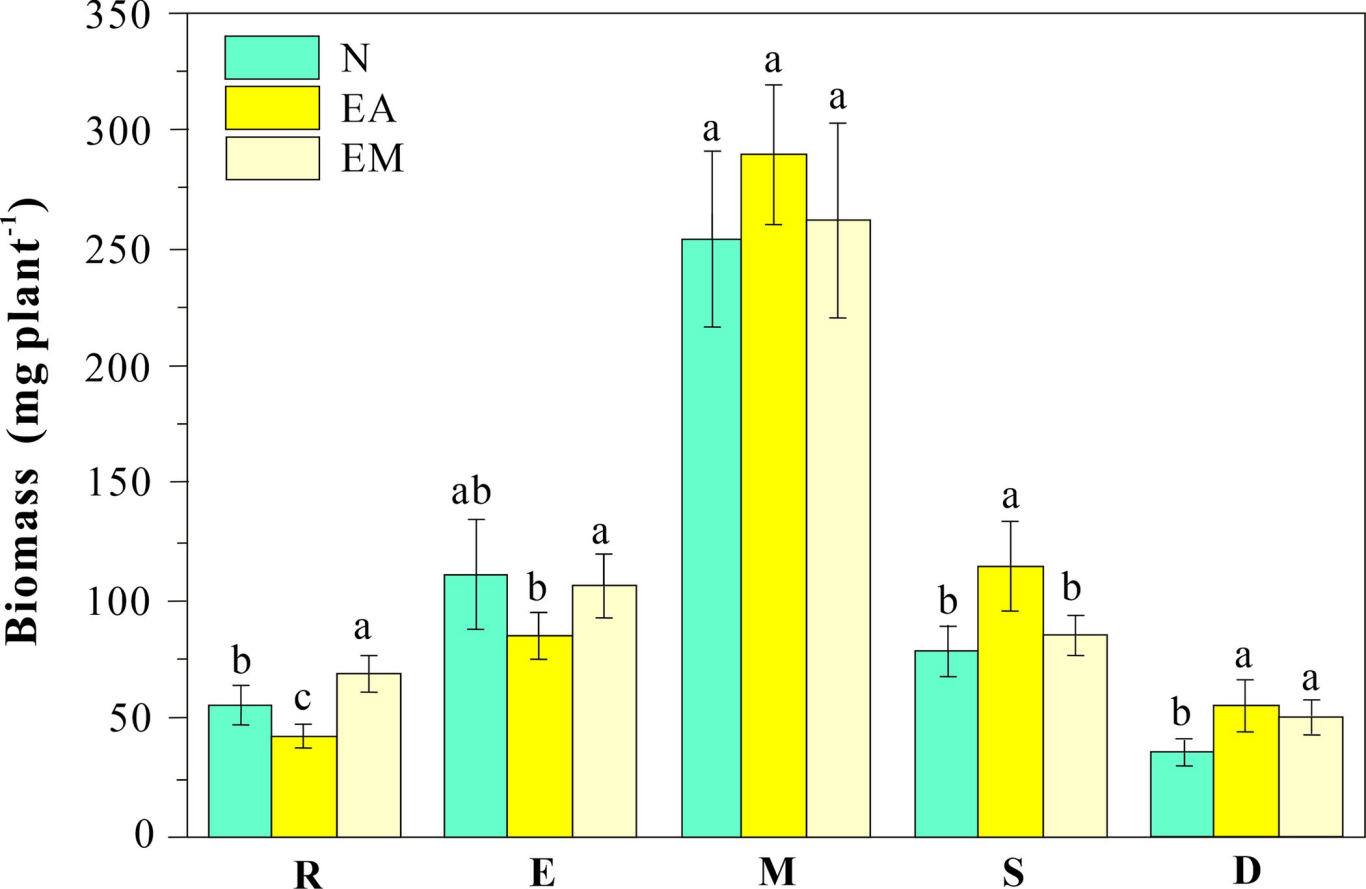
Plant dry weight under various treatments. R, E, M, S, and D represent roots, emerging, mature, senescent, and dead leaves, respectively. N represents normal condition (control), EA represents earthworm activities, and EM represents earthworm mucus. Alphanumeric characters denote results of Fisher’s LSD post-hoc tests. Fig 2 share the same legend. Figs 1 and 2 share the same legend.

*Festuca arundinacea* grew well during the experiment without any visible phytotoxic symptoms. As shown in Fig 1, EA increased the dry weights of mature, senescent, and dead leaves by 14.1, 45.6, and 54.3%, respectively, but decreased the dry biomass of roots and emerging leaves by 24.1 and 23.5%, respectively. The results demonstrated that Earthworm activity (EA) notably influenced the developmental stages of *F*. *arundinacea* leaves, with a noticeable decrease in the biomass of emerging leaves and an increase in the biomass of mature and senescent leaves, indicating an accelerated metabolic rate. Although earthworm activity (EA) significantly reduced the biomass of roots and young leaves compared with the control, the increase in other tissues offset the decrease in roots and emerging leaves. The highest whole plant biomass was observed in the EA group. EM increased the biomass of roots and mature, declining, and dead leaves by 23.4, 3.6, 8.2, and 41.7%, respectively, and reduced the dry weight of emerging leaves by 5.1%. These results indicated that EM had the same effect as EA on the growth rate of *F*. *arundinacea*, particularly on dead leaves.

In the present study, *F*. *arundinacea* generated root and shoot weights similar to those cultivated under other external stresses. Nosalewicz et al. [22] revealed the mechanisms of *F*. *arundinacea* response to drought and reported that the species demonstrated improved functioning under water-deficit conditions during an earlier growth period. In the 50-day experiment, *F*. *arundinacea* generated 43 mg of root dry weight. Zhuo et al. [55] assessed the toxic effects of Cd on *F*. *arundinacea* using four Cd^2+^ treatment regimens (0, 50, 300, and 500 mg L^-1^). The species produced 41 and 420 mg of dry biomass of roots and shoots, respectively, under control (clean soil) conditions, and these dry weights were reduced by 48.2 and 51.2%, respectively, when the Cd content increased to 50 mg L^-1^. These results indicated that high Cd concentrations or bioavailability in spiked soil resulted in phytotoxicity to *F*. *arundinacea*.

Earthworms can consume both dead and living roots [56], which could explain the low root weight of EA-treated *F*. *arundinacea*. Although the application of earthworm mucus led to an increase in plant root biomass compared with the control (Fig 1), the beneficial effect of earthworm activity on root growth did not surpass the loss caused by earthworm consumption by the roots. Wu et al. [57] also documented that the root biomass of *Jatropha curca* increased with increasing density of earthworms (*Eisenia fetida*) until it reached 60 individuals/kg of soil and then decreased significantly, supporting the results of this study. Therefore, the optimal earthworm density for real-scale phytoremediation should be a balance between biomass consumption and stimulating effects.

Earthworms, through their activities, can indirectly enhance plant development by modifying the soil’s physical and chemical properties. Earthworm activity enhances soil porosity and aeration, which in turn can increase root growth and facilitate greater metal uptake. Earthworm mucus has been shown to alter the soil pH and increase the solubility of heavy metals, making them more readily available to plants [58]. Furthermore, earthworm burrowing activities improve the soil structure, leading to enhanced root penetration and access to metals [50]. Our findings suggest that the presence of earthworms and their mucus significantly increases Cd accumulation in *F*. *arundinacea*, likely because of their combined effects on soil properties and plant physiology. Earthworm burrows have been associated with facilitating preferential flow of water and solutes under field conditions [52, 59]. Lipiec et al. [52] studied the microenvironment associated with the activities of *Aporrectodea caliginosa* and *Allolobophora chlorotica* and reported that the drilosphere soil exhibited notably elevated concentrations of nitrate (NO_3_^-^) and ammonium (NH_4_^+^) compared with soil unaffected by earthworm activities. Sheehy et al. [60] assessed the mediating effects of earthworms on the redistribution of C and N in silt-based no-till soils and estimated that a single *Lumbricus terrestris* can deposit approximately 14 mg of nitrate in its burrow. Agapit et al. [61] studied the interactions between earthworm activities and plant growth and reported that *Apporectodea caliginosa* increased thin root elongation and branching density of *Brachypodium distachyon* L. and accelerated the aging of plant leaves by secreting phytohormones such as auxins, cytokinins, and gibberellins, explaining why significantly higher proportions of declining and dead leaves were observed in EA and EM treatments than in the control. However, it is important to note that while the addition of earthworm mucus (EM) to the soil can replicate the effects of earthworm activity (EA) to some extent, the continuous presence and activity of earthworms in the soil are likely to produce more pronounced effects on plant growth and leaf aging because of dynamic interactions not fully mimicked by mucus alone.

### 3.3 Impact of earthworm activity on Cd distribution in different *Festuca arundinacea* leaf types

**Fig 2:**
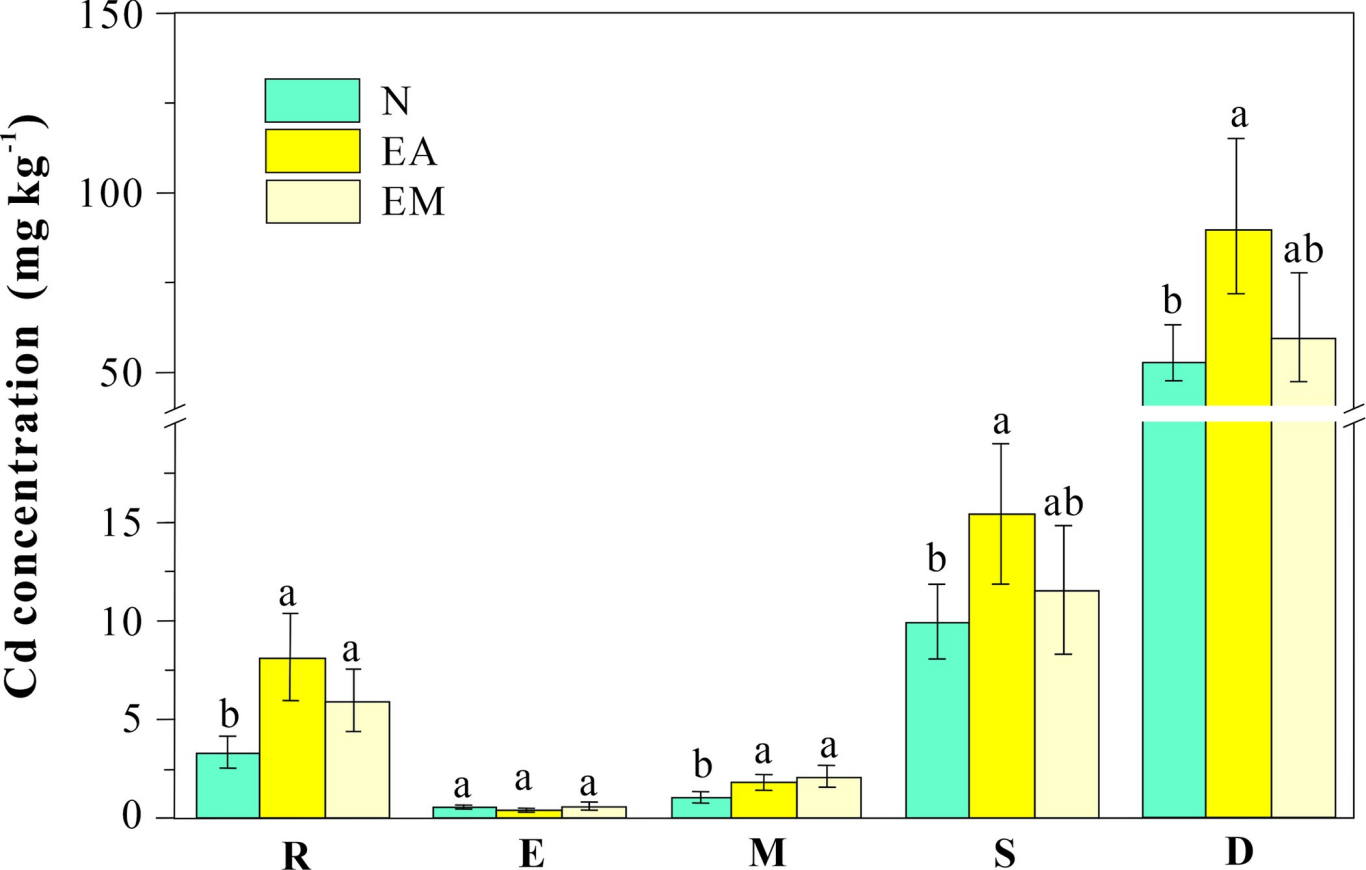
Cd concentrations in different plant parts under various treatments.

Among all the treatments, dead leaves exhibited the highest Cd content, followed by declining, mature, and young leaves (Fig 2). Dead leaves in EA had a significantly higher Cd content than those in EM and the control. Earthworm activity appeared to channel more Cd into declining and dead leaves, as evidenced by the significantly greater cumulative Cd mass in the declining and dead leaves than in other plant tissues (Table 2). The BCF values of Cd in the control plant roots and young, mature, declining, and dead leaves were 0.8, 0.1, 0.3, 2.4, respectively. These results indicate that *F*. *arundinacea* prevents the accumulation of Cd in younger tissues and routes it to older leaves. EA and EM did not affect the accumulation of Cd in the emerging leaves, as exhibited by the identical BCF values (0.1) of the metal in these leaves under all treatments. Nevertheless, the effect of earthworm activity on the BCF of Cd in dead leaves was notable, with a marked increase from 13.1 in the control to 22.2 in the EA plants. Given these observations, it is evident that the presence of earthworms not only influences Cd distribution within plant tissues but also enhances the overall phytoremediation capabilities of *F*. *arundinacea*, particularly by channeling more Cd into decaying plant tissues within pots where their activities are present.

**Table 2:** Phytoremediation parameters of Cd in different plant parts under various treatments.

	Treatments	Root	Young	Developed	Declining	Expired
**Bioaccumulation factors**	N	0.8 ± 0.2 b	0.1 ± 0.02 a	0.3 ± 0.1 b	2.4 ± 0.5 a	13.1 ± 4.2 a
	EA	1.9 ± 0.3 a	0.1 ± 0.02 a	0.4 ± 0.1 ab	3.6 ± 1.0 a	22.2 ± 5.6 a
	EM	1.4 ± 0.4 a	0.1 ± 0.03 a	0.5 ± 0.1 a	2.7 ± 0.8 a	14.8 ± 5.2 a
**Translocation factors**	N	-	0.2 ± 0.03 a	0.4 ± 0.1 a	3.0 ± 0.5 a	16.7 ± 3.1 a
	EA	-	0.1 ± 0.03 b	0.2 ± 0.0 b	1.9 ± 0.5 b	11.5 ± 2.2 b
	EM	-	0.1 ± 0.02 b	0.4 ± 0.1 a	2.0 ± 0.4 b	10.5 ± 2.9 b
**Accumulation capacity** **(μg)**	N	5.6 ± 0.9 b	2.0 ± 0.5 a	9.1 ± 1.8 b	23.5 ± 5.6 b	59.1 ± 11.7 c
	EA	10.4 ± 2.7 a	1.0 ± 0.2 b	15.6 ± 3.7 a	52.9 ± 13.7 a	153.9 ± 28.5 a
	EM	12.2 ± 3.9 a	1.9 ± 0.7 a	16.6 ± 5.2 a	29.6 ± 7.2 b	94.3 ± 26.3 b

Building upon the foundational understanding of Cd distribution influenced by earthworm activity, we further explored Cd content and translocation factors (TF) across different leaf types, underscoring the nuanced effects of earthworm presence and mucus application on *F*. *arundinacea*. When considering different types of leaves as a whole, in N, EA, and EM treatments, the Cd content in all leaves in the whole plant were 6.5, 13.7, and 9.4 mg kg^-1^ based dry mass, respectively. The TFs of Cd in the corresponding treatments were 1.9, 1.7, and 1.6, which were not significantly different. In the control, the TF values calculated separately for young, mature, declining, and dead leaves were 0.2, 0.4, 3.0, and 16.7, respectively. For the EA treatment, the TF values of Cd in young and mature leaves decreased from 0.18 and 0.36 to 0.05 and 0.22, respectively, indicating that earthworm activities can help *F*. *arundinacea* to protect its photosynthetic organs from Cd toxicity more effectively. As shown in Table 2, earthworm mucus (EM) can generate similar effects, but with less magnitude than earthworm activities, on Cd redistribution in various leaf types.

The concentrations of Cd in all plant tissues did not align with the criteria set for Cd hyperaccumulators, which is typically 100 mg kg^-1^ [62]. Furthermore, the distribution pattern of Cd within *F*. *arundinacea* differed from that typically observed in hyperaccumulating plants. Perronnet et al. [63] documented that Cd was distributed unequally within *N*. *caerulescens* and that its concentration increased as senescence occurred. In addition, Wei et al. [64] suggested that *Solanum nigrum* L. accumulates half the total amount of Cd in its cotyledons using ^109^Cd. These results indicate that hyperaccumulators tend to distribute accumulated metals into young leaves. In contrast, non-hyperaccumulators, such as *Ligustrum vicaryi* [65], *Spartina alterniflora* (Negrin et al., 2019 [66]), and *Triticum aestivum* L. Shi et al. [67] have a propensity to accumulate toxins in their senescent leaves. The Cd distribution patterns observed in *F*. *arundinacea* aligned with those commonly found in non-hyperaccumulator plants.

Bagheri et al. [68] introduced the concept of the transpiration stream concentration factor to elucidate the probability of the migration of a particular element to plant leaves. Their research revealed that pollutants have the potential to be transported from the substrate to plant leaves via transpiration, particularly in cases involving solutes with limited soil-particle binding. Manousaki and Kalogerakis [69] found that a high transpiration rate resulted in a higher water uptake capacity in *Tamarix smyrnensis* grown in saline soil and consequently increased metal flux into plant leaves. Wan et al. [70] found that *Pteris vittata* L. cultivated in humid environments displayed a 40% increase in the transpiration rate. Consequently, this higher transpiration rate resulted in a 40% increase in the As content within its aerial parts compared to that when it was grown in dry conditions.

Previous research has established that earthworm utilization can enhance the transpiration rate of plants. Kaur et al. [71] reported that during a 60-day experiment, *Brassica juncea* experienced a 23% reduction in the transpiration rate when subjected to Cd stress (0.5 mM). However, the presence of *Eisenia fetida* (*E*. *fetida*) led to an 11% increase in the transpiration rate of the plant, effectively alleviating the toxicity of the metal to the species. Zhang et al. [72] found that under high salt stress, the inoculation of *E*. *fetida* increased the transpiration rate of *Zea mays* L. significantly, from 0.35 to 0.66 mmol H_2_O m^-2^ s^-1^, and consequently enhanced the salt resistance of the species. Santana et al. [35] evaluated the effects of *Eisenia andrei* on the phytoextraction effect of *Canavalia ensiformis* on Cu and observed an increase in the transpiration rate by 33.5% after the inoculation of earthworms, resulting in significantly higher Cu phytoextraction.

In addition to affecting transpiration, earthworms also affect the speciation, redistribution, and mobility of metals within soils by modifying the levels of dissolved organic carbon (DOC) in soil solutions [73]. In addition, Zhang et al. [74] found that earthworms mobilized Cd by mixing and comminuting soil particles, organic acids, and humic materials. Their assessment of the effects of *E*. *fetida* on metal bioavailability in soils polluted with multiple metals revealed that colonization by native earthworms increased the proportion of Cd in the soluble pool by 27%. Furthermore, earthworms induce plants to generate organic acids that act as carriers of various ions from the roots to shoots [75]. Hence, earthworms can enhance the movement of Cd into the declining tissues of *F*. *arundinacea* by elevating the bioavailability and transpiration rate of the plant.

### 3.4 Enhanced phytoremediation of Cd by *Festuca arundinacea* under various treatments

The accumulation capacity of *F*. *arundinacea* for Cd was used to estimate the phytoextraction effect of the plant. Dry biomass (belowground and aboveground parts) multiplied by Cd concentrations in the corresponding plant tissue provides a measure of the Cd accumulation capacity (AC) of the species. The excess amount of Cd required for removal was calculated by multiplying the difference between the measured Cd concentration (4.2 mg kg^-1^) and the safe limit for agricultural soil (0.3 mg kg^-1^) by the mass of the substrate in each pot (6 kg).

The AC of Cd were 0.19, 0.07, 0.30, 0.78, and 1.97 μg in roots, young, mature, declining, and dead leaves, respectively (Table 2). EA increased the AC of Cd in the roots and mature, declining, and dead leaves by 86.4, 71.2, 125.0, and 160.3%, respectively, while concurrently reducing the Cd mass in emerging leaves by 49.0%. EM also increased the Cd accumulation ability of various plant organs, but to a lesser degree, and this enhancement did not lead to a statistically significant difference compared to the control. Based on the AC value and the relatively high Cd content of the soil, it will take 236, 250, or 283 harvesting cycles to reduce the soil Cd content from 4.2 mg kg^-1^ to 0.3 mg kg^-1^ by harvesting the whole plant, all leaves, or only declining and dead leaves, respectively (Table 3). The EA reduced the number of required cycles by 58.0, 57.5, and 60.0%, respectively. The EM also reduced the number of harvesting cycles by 34.1, 35.7, and 33.3%, respectively. It is noteworthy that the declining and dead leaf harvesting strategy will require 48 more cycles to remove the excess Cd under control, while earthworm activities can decrease from 48 cycles to 13 cycles. If the proportion and Cd content of falling leaves remain consistent throughout the year, the declining and dead leaf harvesting strategy associated with earthworm activities (EA) takes only three more years to clean the soil compared with the whole-plant harvesting strategy, considering the growth period of *F*. *arundinacea*.

**Table 3:** Harvesting cycles required to decontaminate excessive soil Cd.

	Whole plant harvesting	All leaves harvesting	Falling leaves harvesting
**N (cycles)**	236	250	283
**EA (cycles)**	101	105	113
**EM (cycles)**	152	165	189

Complete phytoremediation generally involves soil site preparation, plant growth, transplanting, irrigation, fertilization, harvesting, and replanting. Qu et al. [76] documented that the cost of phytoremediation for Cd was approximately $ 395 per hectare, based on the expenditures of the above steps. All procedures should be repeated after a typical phytoremediation process; however, the cost of soil preparation, plant growth, transplanting, and replanting can be reduced if only the declining and dead leaves of a perennial species such as *F*. *arundinacea* need to be harvested. In addition, plant residues must be managed after phytoremediation. Currently, the feasible methods include combustion and compaction. Some harvested residues with high metal content cannot be used for composting. Although significantly lower than that of traditional methods, the expenditure on phytoremediation is still unacceptable, considering the extensively contaminated areas in China. If a declining and dead leaf harvesting strategy can be applied, the volume of residues can be reduced significantly. In this study, *F*. *arundinacea* stored over 80% of the Cd in its senescent tissues, even though these tissues comprised only approximately 20% of the total plant biomass. After inoculation with earthworms, the declining and dead leaves of the plants accumulated a significantly higher mass of Cd. The declining and dead leaf harvesting strategy associated with earthworm activity can reduce phytoremediation costs by 80%.

Because *F*. *arundinacea* tends to accumulate Pb, Cu, and Cr in its roots and only transfers Cd to its shoots [77], this technology is unsuitable for remediating soils polluted with multiple metals. However, compared with other hyperaccumulators that mobilize almost all metals in the soil but only specific metals, *F*. *arundinacea* efficiently extracted Cd without inducing the mobilization of other soil metals, thereby decreasing the leaching risk in the soil remediation procedure. We also know that the amount of mucus excreted from earthworms in EA is not equal to the amount of mucus applied in EM. The treatments were designed to confirm the effects of earthworm activities and earthworm mucus on the development and Cd phytoextraction capacity of *F*. *arundinacea*, and to evaluate whether earthworm mucus can replace earthworm inoculation in future phytoremediation. Both these methods can improve phytoremediation.

Although the status of earthworms at the conclusion of the experiment was not systematically recorded, it is important to note that the population of earthworms within EA increased beyond the initial count of 30. This is attributable to the strong reproductive capacity of earthworms. The increase in earthworm population likely contributed to the enhanced effects observed in EA compared to EM, where the assumption was of a constant earthworm population based on the mucus collected from 30 earthworms. Future studies should aim to precisely quantify the earthworm population dynamics and their implications for the effectiveness of phytoremediation.

## 4. Conclusion

Earthworm activities significantly enhanced the phytoremediation capabilities of *F*. *arundinacea* in Cd-contaminated soils, notably by increasing both the biomass yield and Cd concentration in declining and dead leaves. Following earthworm inoculation, the Cd concentration in these leaves significantly increased, directing over 85% of the plant’s total Cd content to the senescent tissues, which comprised only 30% of the plant biomass. This strategic accumulation points to a promising avenue for optimizing phytoremediation efforts through selective harvesting, potentially substantially reducing costs. Additionally, the findings indicate that, while earthworm mucus can replicate the effects of live earthworm activities to some extent, the presence of live earthworms notably enhances Cd translocation and accumulation in senescent tissues, offering a superior phytoremediation mechanism. These observations contribute to the understanding of the roles of biological agents in enhancing the efficacy of heavy metal soil remediation, providing a sustainable strategy to amplify phytoremediation effectiveness.

## Supporting information

S1 Data (XLSX)
